# Proteomic Analysis of the Low Mutation Rate of Diploid Male Gametes Induced by Colchicine in *Ginkgo biloba* L.

**DOI:** 10.1371/journal.pone.0076088

**Published:** 2013-10-22

**Authors:** Nina Yang, Yuhan Sun, Yaru Wang, Cui Long, Yingyue Li, Yun Li

**Affiliations:** 1 National Engineering Laboratory for Tree Breeding, Key Laboratory of Genetics and Breeding in Forest Trees and Ornamental Plants of the Ministry of Education, College of Biological Sciences and Technology, Beijing Forestry University, Beijing, China; 2 Shijiazhuang Pomology Institute, Hebei Academy of Agriculture and Forestry Sciences, Shijiazhuang, China; Pennsylvania State University, United States of America

## Abstract

Colchicine treatment of *G. biloba* microsporocytes results in a low mutation rate in the diploid (2n) male gamete. The mutation rate is significantly lower as compared to other tree species and impedes the breeding of new economic varieties. Proteomic analysis was done to identify the proteins that influence the process of 2n gamete formation in *G. biloba*. The microsporangia of *G. biloba* were treated with colchicine solution for 48 h and the proteins were analyzed using 2-D gel electrophoresis and compared to protein profiles of untreated microsporangia. A total of 66 proteins showed difference in expression levels. Twenty-seven of these proteins were identified by mass spectrometry. Among the 27 proteins, 14 were found to be up-regulated and the rest 13 were down-regulated. The identified proteins belonged to five different functional classes: ATP generation, transport and carbohydrate metabolism; protein metabolism; ROS scavenging and detoxifying enzymes; cell wall remodeling and metabolism; transcription, cell cycle and signal transduction. The identification of these differentially expressed proteins and their function could help in analysing the mechanism of lower mutation rate of diploid male gamete when the microsporangium of *G. biloba* was induced by colchicine.

## Introduction

*Ginkgo biloba* L. is a living fossil tree with no close living relatives. It is cultivated because of its importance in traditional medicine and food value; and as an ornamental tree for its attractive shape and foliage. Recent studies have also implicated a use for Gingko extract in prevention of Alzheimer's disease [1] and dementia [2], thus creating a high market demand for this tree species. However, it has a tedious and inefficient growth process, and although effective breeding methods are emerging, it is still a challenge to meet the increased production requirement. Gingko extract contains secondary metabolites that have medicinal value; therefore, breeding methods that help in enhancing the quantity of these secondary metabolites are desirable. Since triploid (3n) plants have fast growth and a higher content of secondary metabolites as compared to 1n and 2n, ploidy breeding can be a useful tool for breeding new economic varieties of *G. biloba*.

Triploid plants are produced when diploid male gametes are induced and fertilized with normal female gametes. Colchicine is a wide-ranging and effective triploid-inducer used in studies of polyploidy breeding. It hinders the formation of spindle fibers in the metaphase of cell division and plays a minor role in affecting the structure of chromosomes [3].

Previous studies have shown that diploid pollen could be produced from the microsporocytes treated with colchicine, however, the rate of mutation was only 7 per cent [4] as compared to other trees such as, white *Populus* –88% [5], *Eucommia-* 49.5% [6], and black *Populus* −90.25% [7]. The low mutation rate in diploid male gametes of *G. biloba* is insufficient for its application in ploidy breeding.

Sun et al. [8] showed that the microsporangium and microsporocyte wall are not the major factors hindering the process of colchicine induction of diploid male gametes in *G. biloba*. Several other studies have indicated that the proteome is an influencing factor in the formation process of male gametes. The events of meiosis are controlled by a protein enzyme complex known as the “maturation promoting factor.” These enzymes interact with one another as well as with other cell organelles to cause the breakdown and reconstruction of the nuclear membrane, the formation of the spindle fibers, and the cell division process [9]–[12].

We hypothesize that perhaps there are some proteins that do not destroy the chemical structure of colchicine; however, they can interfere with the process in which tubulin combines with colchicine. Therefore, it is necessary to determine differences between protein function in colchicine-treated and untreated microsporocytes during meiosis. Since colchicine stress has not yet been extensively examined at the proteome level in plant breeding studies, no data on this topic are currently available in the literature. Therefore, this study provided a framework to investigate the cause of low-level diploid male gametes formation in *G. biloba* treated with colchicine.

## Materials and Methods

### Chemicals and Reagents

IPG strip (17 cm, pH 4–7), 2-DE marker, Biolyte (pH 4–6 and pH 5–7), mineral oil, Dithiothreitol (DTT) and iodoacetamide were obtained from Bio-Rad Laboratories (Hercules, CA). Tris-base, ammonium persulfate (AP), agarose, sodium dodecyl sulfate (SDS), N,N,N',N'- tetramethylethylene diamine (TEMED), glycin, Acrylamide, N,N'-methylenebisacrylamide, bromophenol blue, Coomassie brilliant blue (CBB) G-250, thiourea, 3-[(3-cholamidopropyl)-dimethylammonio]-1-propane sulfonate (CHAPS), urea, glycerol, and bovine serum albumin (BSA) were obtained from Sigma (St. Louis, MO). Trypsin was obtained from Roche (city, state of manufacturer); trifluoroacetic acid (TFA) and acetonitrile were from JT Baker (Phillipsburg, NJ).

### Plant materials

The male floral branches of *G. biloba* were collected in the spring season from the Teaching and Experimental Forestry Center of Beijing Forestry University (40.04060 N, 116.05256E). The branches were placed in cold storage (4°C) for 24 h before the microsporangiums of *G. biloba* began to develop in meiosis. The branches were cultured in the greenhouse (25°C) in order to induce meiosis. The microsporangia of *G. biloba* were treated with colchicine solution as soon as most of them turned green, were full in granule and the top bud scales began to open. Colchicine was applied using cotton balls soaked in 0.8% (8 mg/mL) colchicine solution [4]; the control samples were treated with cotton balls soaked distilled water only. Three independent biological replicates of each sample were taken. The samples were collected 48 h post treatment.

### Protein Extraction

Total protein extracts were prepared according to the method described by Wang et al. [13] with minor modifications. The powder of the microsporangia of *G. biloba* was suspended in 1 mL cold extraction solution (10% TCA in acetone containing 1 mM PMSF and 0.07% β-mercaptoethanol) and then incubated at –20°C for 12 h. The samples were then centrifuged at 12,000×g for 30 min at 4°C. The pellets were washed thrice with prechilled acetone containing 0.07% β-mercaptoethanol, and kept at –20°C for 1 h before centrifugation at 12 000×g for 30 min at 4°C, and then dried under vacuum for10 min at 4°C.

The dried protein pellets were dissolved in lysis buffer containing 7 mol/L urea, 2 mol/L thiourea, 4% (w/v) CHAPS, 65mmol/L DTT, 0.2% (v/v) carrier ampholytes (0.1% pH = 5–7, 0.1% pH = 4–6) and incubated at room temperature for 2 hours and then centrifuged at 12 000×g for 30 min at room temperature. The protein concentration of the supernatant was determined by Bio-Rad Protein Assay Kit II (manufacturer, city, state). The supernatant was loaded for isoelectrofocusing.

### 2-D Electrophoresis

Two-dimensional gel electrophoresis was performed according to the Bio-Rad handbook and the procedure developed by Gorg et al. [14]. 700 μg of the sample was loaded onto analytical as well as preparative gels. For IEF, the Bio-Rad Mini-PROTEAN II 2-D system and pH 4–7 IPG strips (17 cm) were used according to the manufacturer's recommendations. The IPG strips were rehydrated for 14 h in 350 μL rehydration buffer containing protein samples. The images were taken as described by Wang et al. [13].

The gel strips were equilibrated for 15 min with two equilibration buffers: (i) reducing buffer for 15 min (50 mM Tris-HCl buffer, pH 8.8, 6 M urea, 20%v/v glycerol, 2% w/v SDS, and 1% w/v DTT). (ii) alkylating buffer for 15 min (50 mM Tris-HCl buffer, pH 8.8, 6 M urea, 20%v/v glycerol, 2% w/v SDS, and 2.5%w/v iodacetamide). SDS-PAGE was performed with 12% gels using the PROTEAN II xi Cell system (Bio-Rad, Hercules, CA, USA). The gels were run at 0.5 h at 1 W per gel, then at 8 W per gel until the dye front reached the gel bottom. The separated proteins were visualized by CBB G250 staining.

### Image Acquisition and Data Analysis

The 2-DE images were acquired with an Image Scanner GS-800 (Bio-Rad, Hercules, CA, USA) in transmission mode. The image analysis was carried out by a combination of manual visualization and software calculations using PDQuest 8.0.1 Gel Analysis Software (Bio-Rad, Hercules, CA, USA). In order to correct the variability due to CBB-staining and to reflect the quantitative variations in intensity of protein spots, the spot volumes were normalized as a percentage of the total volume in all of the spots present in the gel. All 2-DE images were globally analyzed by the software and the identified spots were manually rechecked. Significantly different spots were drawn from a comparison of any two gels.

### Tryptic Digestion

The CBB stained spots were excised from the 2-DE gels and destained for 30 min ×3–4 times until the gel was transparent with no color, using a destaining solution consisting of 50% acetonitrile and 25 mM ammonium bicarbonate and then they were immersed in acetonitrile (100%) for 10 min. The gels were dried for 30 min using a Speed-Vac system. 2.5 mL of 25 mM (NH_4_)HCO_3_ was added to 25 μg of trypsin (final concentration 10 ng/μL); 10 μL of this trypsin solution was pipetted on each dried protein spot and the sample was incubated at 4°C for 60 min. The supernatant was discarded to minimize autodigestion of trypsin. Then the Ep tubes were placed upside down and incubated at 37°C for 14 h. To extract the peptide fragments from the tryptic digest, 20 μL of 5% (v/v) TFA was added to the digest and the sample was incubated at 37°C for 60 min, then the supernatant was transferred into another Ep tube. Thereafter, 20 μL of 50% (v/v) acetonitrile [containing 2.5% (v/v) TFA] was added to the gel and the sample was incubated at 30°C for 60 min. The supernatants were pooled together and dried for 2 h heating in a Speed-Vac system.

### Protein identification by MALDI-TOF/TOF-MS

Before obtaining the mass spectra of the peptide mixture, the digested peptides were desalted and cleaned with ZipTip C18 pipette tips (Millipore Corporation, Bedford, MA, USA) according to the manufacturer's instructions. All analyses were performed using a Bruker Daltonics Autoflex (Bruker Daltonics Billerica, MA, USA) operated in the delayed extraction of 190 ns and reflector mode with an accelerating voltage of 20 kV. The peptide mixture was analyzed using a saturated solution of R-cyano-4-hydroxycinnamic acid (CHCA Bruker Daltonics Billerica, MA, USA) in 50% acetonitrile/0.1% trifluoroacetic acid. External calibration was performed with a peptide calibration standard (Bruker Daltonics Billerica, MA, USA, Part No.: 206195) and internal calibration with trypsin autoproteolytic fragments. The samples were analyzed on a MALDI-TOF/TOF-MS 4800 proteomics analyzer (Aglient, USA) and data were analyzed using GPS explorer software (Applied Biosystems, Foster City, Calif.) and MASCOT software (Matrix Science, London, UK).

## Results

### Comparative protein profiles of colchicine treated and control microsporangia

**Figure 1:**
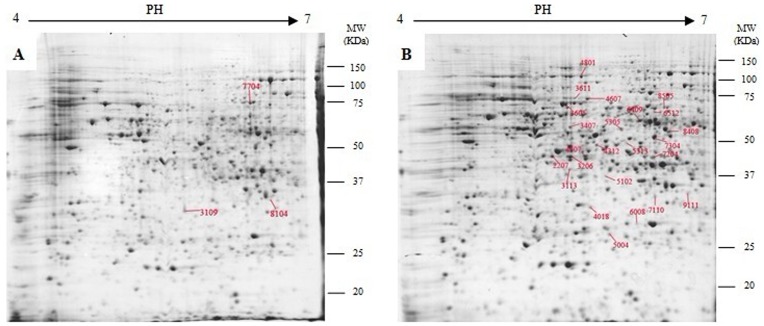
Representative 2-DE gels of untreated (A) and colchicine treated (B) microsporangia. Twenty-seven of the spots showing at least a 2-fold change with *p*<0.05 were analyzed by MALDI-TOF/TOF-MS.

Approximately, 1000 microsporangia proteins were detected on coomassie brilliant blue-stained gels (Figure 1B). To accurately and quantitatively analyze proteomic changes, spot volume differences of more than 2-fold between two identical spots were defined as significant. The spots that revealed changing their abundance significantly after quantitative image analysis were randomly selected to check again. A total of sixty-six proteins were differentially expressed in response to colchicine (Figure 1A, 1B). It was noted that some protein spots demonstrated qualitative changes in intensity. The spots 7704, 8104 and 3109 were absent in the colchicine-treated gels (Figure 1).

### MOLDI-TOF/TOF-MS identification and classification of colchicines-responsive proteins

Thirty-nine of these differentially expressed spots were arbitrarily selected and excised from the gels and subjected to MOLDI-TOF/TOF-MS. Twenty-seven proteins were successfully identified and are listed in Table 1.

**Table 1:** Obs. pI and Mr, observed pI, Mr calculated from the 2D-gels with PDQuest 8.0.1 software according to standard marker proteins.

Ssp	Name	Accession No	Protein Score	Mr Theor./obs.	pI Theor./obs.	Expression pattern
**Proteins involved in ATP generation, transport and carbohydrate metabolism**						
3407	ATP synthase beta subunit [Triticum aestivum]	gi|525291	211	59.21/71.50	5.56/5.58	up
4018	thiazole biosynthetic enzyme [Pseudotsuga menziesii]	gi|56481847	118	37.30/34.22	5.85/5.44	up
7204	glutamate-1-semialdehyde 2,1-aminomutase [Nicotiana tabacum]	gi|19875	118	50.98/55.18	7.05/6.47	up
7304	isocitrate dehydrogenase (NADP+) [Arabidopsis thaliana]	gi|15221788	85	47.20/65.31	7.58/6.37	up
5305	enolase2 [Zea mays]	gi|162460735	182	48.13/68.08	5.70/6.03	down
3113	Cytochrome c[Ginkgo biloba]	gi|117987	54	123.5/48.10	9.76/5.14	down
3109	33 kDa oxygen-evolving protein [Arabidopsis thaliana]	gi|22571	142	35.11/35.08	5.68/5.31	disappear
7704	putative transketolase [Oryza sativa Japonica Group]	gi|28190676	103	79.98/94.68	6.12/6.30	disappear
8104	NADPH thioredoxin reductase [Arabidopsis thaliana]	gi|468524	73	32.42/38.43	5.78/6.60	disappear
**Protein metabolism**						
3605	heat shock protein 70 like protein [Arabidopsis thaliana]	gi|4467097	84	71.13/88.74	5.31/5.54	up
3611	heat shock protein 70 [Cucumis sativus]	gi|6911553	123	70.00/92.07	5.29/5.63	up
8408	26S proteasome ATPase subunit [Pisum sativum]	gi|49175787	214	23.56/68.35	7.16/6.69	up
4207	Protein disulfide-isomerase [Medicago truncatula]	gi|357442333	80	40.47/55.98	5.54/5.81	down
4312	Elongation factor Tu [Chlamydomonas reinhardtii]	gi|41179007	182	45.71/61.41	5.90/5.89	down
5313	Elongation factor Tu [Arabidopsis thaliana]	gi|15236220	112	49.38/62.11	6.25/6.18	down
**ROS scavenging and detoxifying enzymes**						
5004	GST [Ginkgo biloba]	gi|66736578	421	25.77/26.64	6.24/5.71	up
6008	ascorbate peroxidase [Ginkgo biloba]	gi|220898265	296	27.51/30.31	5.81/6.29	down
9111	ferredoxin-NADP + reductase [Arabidopsis thaliana]	gi|162459168	98	39.30/40.13	8.53/8.66	up
**Wall remodeling and metabolism**						
7110	2-dehydro-3-deoxyphosphooctonate aldolase [Arabidopsis thaliana]	gi|13620976	65	31.60/37.81	6.33/6.38	up
2207	golgi associated protein se- Zea mays]	gi|162463414	124	41.18/57.79	5.75/5.38	down
**Transcription, cell cycle and signal transduction**						
4607	ARF-L1 protein [Ginkgo biloba]	gi|291196881	40	110.7/89.10	5.64/5.45	up
4801	Cell division cycle protein 48 homolog	gi|1705678	69	89.77/100.8	5.18/5.72	up
8505	putative polyprotein [Nicotiana tabacum]	gi|15963359	65	61.40/85.59	9.36/6.51	up
**Unclassified proteins**						
3206	Os02g0698000 [Oryza sativa (japonica cultivar-group)]	gi|115448091	75	44.84/54.23	5.68/5.14	down
5102	putative protein [Arabidopsis thaliana]	gi|7573371	98	34.75/47.97	6.19/6.00	down
6409	Os02g0125100 [Oryza sativa (japonica cultivar-group)]	gi|115443927	73	55.50/75.83	7.05/6.15	up
6512	Sequence 4 from patent US 6946283	gi|77372606	40	99.47/77.53	5.53/6.36	down

ER: endoplasmic reticulum, PM: Plasma Membrane, M: Mitochondrion.

**Figure 2:**
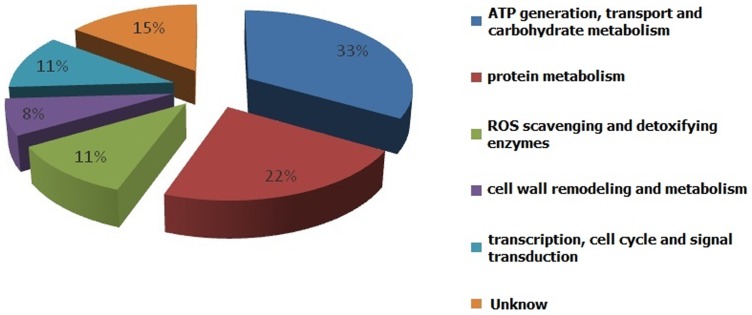
Functional categorization of different proteins. Digitals stand for the protein number of each functional category.

The identified proteins were associated with a wide variety of cellular processes. They were classified into several categories according to their function (Figure 2), including the ATP generation, transport and carbohydrate metabolism, protein metabolism, ROS scavenging and detoxifying enzymes, cell wall remodeling and metabolism, transcription, cell cycle, and signal transduction.

**Figure 3:**
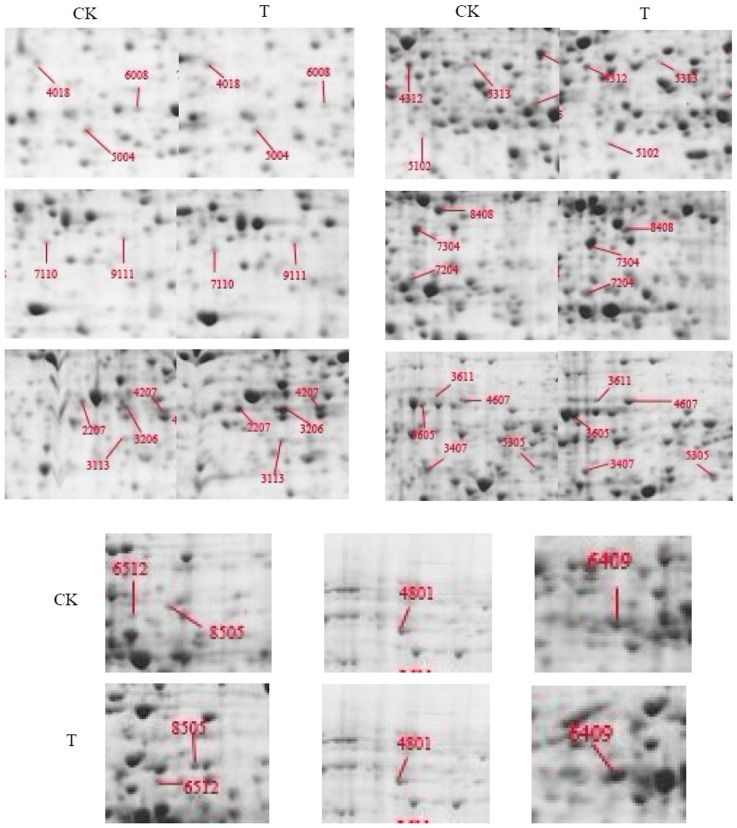
Comparison of the differential proteins expression level in two groups. CK: the control, samples treated with cotton balls soaked distilled water only. T: the test, samples treated with cotton balls soaked 0.8% (8 mg/mL) colchicine solution.

The identified spots corresponded to proteins that were related to energy, carbohydrate metabolism and photosynthesis. In our study, nine proteins were in this category. Four of them were found to be up-regulated, and three of them were changed in the opposite way. The rest of them evenly has been disappeared after colchicine treated. Several chaperones involved in protein processing, folding and degrading were also identified as being responsive to colchicine treatment. These included two HSP (spot3605 and spot3611) and two EF-Tu (spot4312 and spot5313) proteins. In this study, the levels of protein (3605), which was analogous to HSP-70 (3611) and heat shock protein 70, were found to be elevated (Figure 3).

The SE-wap41 (spot 2207) was found to be down-regulated. 2-dehydro-3-deoxyphosphooctonate aldolase (spot 7110), a protein involved in cell wall remodeling and metabolism, was found to be up regulated. Both of them indicating that colchicines might perhaps have interfered with cell wall formation. The three spots, 4607, 4801 and 8505 were related to transcription, cell cycle, and signal transduction. Spot 4607 was found to be a response factor of a plant growth hormone and was up-regulated by colchicine treatment. Spot 4801 was found to be the homolog of CDC48 and spot 8505 was classified as a putative polyprotein; and expression levels of both proteins were elevated in this study.

APX (spot 6008) and GST (spot 5004) were found to be a part of the antioxidant system employed by plants [15]. Previous microarray results demonstrated that these proteins were responsive to various stresses including osmotic, drought, and cold stresses [16]–[18]. We found that the abundance of GST was three times higher post colchicine treatment. However, the abundance of APX was drastically down regulated (half of the level in control).

## Discussion

The protein profiles of microsporangia treated with colchicine enabled us to classify the individual response proteins on the basis of their function.

### 1. Cell wall remodeling and metabolism

The decline in the abundance of se-wap41 after 48 h of colchicine treatment corroborated the results of Chen et al. [19]. Se-wap41, a salt-extractable 41-kD wall-associated protein that has been reported to label plasmodesmata and the Golgi was found to be a class 1 reversibly glycosylated polypeptide (C1 RGP) [9]. Using immunogold labeling, Dhugga et al. [20] showed that RGP1 was specifically localized to Golgi stacks, where it was likely to be involved in xyloglucan biosynthesis [21]. This suggests that colchicine can interfere with the cell wall remodeling. Hogetsu and Shibaoka [22] also demonstrated that colchicine could inhibit cell division and disturb the microfibril arrangement in *Closterium acerosum.*

2-dehydro-3-deoxyphosphooctonate aldolase (spot 7110) is known to play an important role in the LPS biosynthetic pathways. Lipopolysaccharide (LPS) serves as a selectively permeable membrane for organic molecules, increases the negative charge of the cell wall and stabilizes the overall membrane structure. Wang et al. [23] found that 2-dehydro-3-deoxyphosphooctonate aldolase was upregulated in boron deficient *Brassica napus* roots. It is speculated that plants synthesize LPS to resist injury when cell membranes suffer the colchicine stress.

### 2. Proteins involved in ATP generation, transport and carbohydrate metabolism

Respiration is critical to metabolism in higher plants. In addition to its link with carbon metabolism, respiration releases energy stored in carbon-based compounds in a controlled manner for cellular use. It also generates many carbon precursors for biosynthesis. Carbon metabolism enzymes appear to be major targets for oxidative modification and breakdown *in vitro* and *in vivo* [24]. In our study, 48% of the proteins were found to be involved in ATP generation, transport and carbohydrate metabolism. Most of them responded in a manner similar to other abiotic stress conditions. For example, it was noted that enolase (spot5305), which catalyzed the formation of high-energy phosphoenol pyruvate from 2-phosphoglycerate in the glycolytic pathway, decreased after 48 h of colchicine stress. Previous reports showed that a mutation in the enolase locus results in the repression of cold-responsive genes and therefore, it acts as a positive regulator of cold-responsive genes [25]. The decreased abundance of enolase protein was similar to the response of *Arabidopsis* roots to NaCl stress [26]. Other energy-related proteins, including the two ATP synthases (spot 8408 and 3407) were found to be elevated in response to colchicine treatment. This response was similar to that observed in other stress conditions such as salt or cold stress [27]–[29].

Thiazole biosynthetic enzyme (spot 4018) also showed higher abundance in response to colchicine treatmenttreated. Tunc-Ozdemir et al.[30] indicated that when Arabidopsis (*Arabidopsis thaliana*) plants showed an elevated expression of transcripts encoding thiamin biosynthetic enzymes in response to abiotic stress conditions, such as high light, cold, osmotic stress, salinity, and oxidative treatments.

The glutamate 1-semialdehyde 2,1-aminomutase (spot 7204), which is involved in the C5 pathway for synthesis of δ-aminolevulinic acid (ALA) [31]. δ-Aminolevulinic acid (ALA) is the first intermediate in the synthesis of chlorophylls [32].

### 3. ROS scavenging and detoxifying enzymes

Abiotic stresses induce the production of ROS, which can cause damage to cellular components and act as a signaling molecule for stress responses [15]. In recent years, additional reports have indicated that GSTs in plants play major roles in herbicide detoxification [33]–[40]. GSTs also conjugate natural products such as anthocyanins, serving as binding proteins or ligandins for plant hormones, or catalyze GSH-dependent peroxidase and isomerase reactions [34], [41]. Herbicide safeners are chemicals used to minimize the effect of the herbicides on crop plants. They protect cereal crops from herbicide injury by increasing the expression of GSTs as well as other herbicide-detoxifying enzymes and proteins such as cytochrome P-450 s, glucosyl transferases, and tonoplast transporters that can detoxify herbicides and transport them into the vacuole [39]. In this study, colchicine treatment resulted in elevated levels of GSTs, perhaps to reduce colchicine toxicity. An increased detoxification of compounds mediated by high levels of glutathione (GSH) and glutathione transferase (GST) has been found in cells resistant to cochicine [42]. Ruiz-Gomez et al. [42] suggested that GSTs may play an important role in main multidrug resistance (MDR) pumps in cells that express high levels of P-gly-coprotein (P-gly).

APX in plants utilizes ascorbate as an electron donor to catalyze H_2_O_2_ conversion into H_2_O. It is a multigenic enzyme localized in different cellular compartments, which complements and coordinates antioxidant defenses [43]. In rice it was induced under conditions like ozone stress, drought stress, chitosan, and in blast lesion mimic mutants [44]–[47]. Studies on the possible interference of colchicine and H_2_O_2_ in *Arabidopsis thaliana* cv.Col-0 suggested that APX was the most sensitive enzyme to colchicine and H_2_O_2_ [48].

FNR (spot 9111) is a widespread family of FAD-containing enzymes. In different tissues and organisms these flavoproteins catalyze the reversible electron transfer between two molecules of the obligatory one-electron carrier Fd and a single molecule of NADP (H) [49]. The expression of a cloned plant FNR gene in a mutant *E. coli* strain did restore the oxidative tolerance to wild-type levels, indicating that the eukaryotic flavoenzyme behaves as a toxic radical scavenger in the bacterial host [50]. In our study, the FNR (spot 9111) was found to increase under colchicines stress. This indicates the toxic radical scavenger function of FNR in gymnosperms.

### 4. Protein metabolism

The proteolytic system, consuming metabolic energy, plays an essential role in the regulation of cellular functions by catalyzing rapid and irreversible reactions for various important biological processes, such as the cell cycle, apoptosis, signal transduction, protein processing, and immune and stress responses [51]. We detected three proteins that promote the proper folding of proteins and proteolysis and found that their abundance increased following colchicine treatment. One of them was the 26 s proteasome ATPase subunit. G. N. DeMartino and his colleagues [52] attempted the molecular cloning of cDNAs encoding the human 26S proteasome regulatory subunits. These subunits are divided into two distinct sub-groups, ATPase and non-ATPase subunits. The 26S proteasomal regulatory complex has intrinsic ATPase activity that seems to play an essential role in its function [52], [53]. The 26S proteasome attacks poly- or multi-Ub chain to act as a degradation signal for proteolysis. Furthermore, research has shown that different kinds of stresses can result in degradation of the Ub chain. Treatment with HgCl_2_ increases the expression of ubiquitin genes in *Nicotiana;* and a strong accumulation of a ubiquitin conjugating enzyme (Ubc1) was observed in tomato after exposure to CdCl_2_ [54], [55]. The expression of polyubiquitin gene is affected by darkness, UV radiation, starvation, and an enhanced level of ozone [56]–[59]. A significant increase in the level of de novo biosynthesis of ubiquinone and plastoquinone (PQ) in plant tissues was observed as a result of colchicine treatment [60].

One of the first described chaperone regulators of Tau is the Hsp70 chaperone family. Increased levels of Hsp70 promote tau solubility and tau binding to microtubules; the tau protein is normally expressed in the cytoplasm of cell bodies and axons, where it binds to and stabilizes microtubules [61]. The greatly increased abundance of HSP 70 s in our study suggested that the *G. biloba* microsporangia were building resistance to colchicine. This powerful resistance helped in maintaining the stability of microtubule when the microtubule of *G. biloba* was treated by colchicine.

Elongation factor-1α (EF-1α), a highly conserved protein named for its role in protein translation, is also a microtubule-associated protein (MAP) [62] and binds to the microtubule lattice [63]–[65]. This protein can also modulate microtubule dynamics *in vitro* and *in vivo* [66]–[71]. In our study this protein was found to be down regulated. Therefore, *G. biloba* might have a strong self-protection system to defend the colchicine invasion, which hinders the induction of diploid gametes.

### 5. Transcription, cell cycle and signal transduction

Auxin plays a crucial role in diverse aspects of plant growth and development, including tropic responses, apical dominance in the shoot, lateral root formation and differentiation of the vascular system. In this study, the increased ARF-L1protein indicated that colchicine may stimulate the differentiation of microsporangiums.

In particular, studies of cell division cycle protein 48 (Cdc48p) in *Saccharomyces cerevisiae* have uncovered roles in many cellular processes, including membrane fusion [10], ERAD [11] and spindle disassembly [12].

## Conclusion

In this study, *G. biloba* microsporangia were subjected to 48 h of colchicine treatment followed by proteomic analysis of treated and control samples. A total of 27 colchicine-responsive proteins were identified (Table 1) by 2-D gel electrophoresis. Complementary strategies to protein electrophoresis can be employed at the transcript and metabolite levels in future to gain insight into the intricate network of plant responses to colchicine. Proteins are the most direct reflection of coclhicine response. And genes are the most fundamental evidences. Through above analysis, we can see the possible roles the twenty-seven proteins played in defending colchicine invasion. So a new dimension to our work is pointed out. The differently expressed proteins should be the most important part of future study. The real reason for the low mutation rate in diploid male gametes of *G. biloba* might be found out among them. Much work still to be done about the twenty-seven proteins, such as comparing the twenty-seven genes with colchicine responsive genes in other species to see if any of them are specific to *G. biloba*. If we can confirm the significant proteins and find the mechanism of lower mutation rate of diploid male gamete when the microsporangium of *G. biloba* was induced by colchicine, we can try to optimize the ploidy breeding method in *G. biloba*.

## References

[1] Vellas B, Coley N, Ousset PJ, Berrut G, Dartigues JFO, et al (2012) Long-term use of standardised ginkgo biloba extract for the prevention of Alzheimer's disease (GuidAge): A randomised placebo-controlled trial. The Lancet Neurology 11(10): 851–9 10.1016/S14744422(12)702065 22959217

[2] Weinmann S, Roll S, Schwarzbach C, Vauth C, Willich SN (2010) Effects of Ginkgo biloba in dementia: systematic review and meta-analysis. BMC geriatrics 10: 14 10.1186/147123181014 20236541 PMC2846949

[3] Li Y, Feng DL (2005) Advances in Research into Polyploidy Breeding of Woody Plants. Chinese Bulletin of Botany 22 (3): 375–382.

[4] Cheng JX, Li Y, Wang Y, Jiang JZ, Wang Q, et al (2006) Pollen chromosome doubling of Ginkgo biloba induced by colchicines. J Beijing For Univ 28(6): 15–22.

[5] Kang XY, Zhu ZT, Lin HB (1999) Study on the effective treating period for pollen chromosome doubling of Populus tomentosa × P. bolleana. Scientia Silvae Sinicae 35(4): 21–24.

[6] Gao P, Lin W, Kang XY (2004) Pollen chromosome doubling of Eucommia ulmoides induced by colchicine. J Beijing For Univ 26(4): 39–42.

[7] Zhang JF (2006) Populus 2n pollen formation mechanism and induction of technology. PhD thesis. Beijing For Univ Lib 2–10.

[8] Sun YH, Li Y, Yang NN, Sun P, Yuan CQ (2011) Preliminary study on the inducement effect of colchicine during microsporogenesis of Ginkgo biloba L. Afr J Biotechnol. 10(28): 5476–5488.

[9] Sagi G, Katz A, Guenoune-Gelbart D, Epel BL (2005) Class 1 reversibly glycosylated polypeptides are plasmodesmal-associated proteins delivered to plasmodesmata via the Golgi apparatus. Plant Cell 17: 1788–1800 10.1105/tpc.105.031823 15879561 PMC1143077

[10] Latterich M, Frohlich KU, Schekman R (1995) Membrane fusion and the cell cycle: Cdc48p participates in the fusion of ER membranes. Cell 82: 885–893 10.1016/00928674(95)902686 7553849

[11] Rabinovich E, Kerem A, Frohlich KU, Diamant N, Bar-Nun S (2002) AAA-ATPase p97/Cdc48p, a cytosolic chaperone required for endoplasmic reticulum-associated protein degradation. Mol Cell Biol 22: 626–634 10.1128/MCB.22.2.626634.2002 11756557 PMC139744

[12] Cao K, Nakajima R, Meyer HH, Zheng Y (2003) The AAA-ATPase Cdc48/p97 regulates spindle disassembly at the end of mitosis. Cell 115: 355–367 10.1016/S00928674(03)008158 14636562

[13] Wang YR, Li Y, Sun YH, Wang Q (2010) Microspore Protein Extraction from Ginkgo biloba and Optimization of Two-dimensional Electrophoresis Techniques. J Southwest For Univ 30(3): 45–49.

[14] Gorg A, Obermaier C, Boguth G, Weiss W (1999) Recent developments in two-dimensional electrophoresis with immobilized pH gradients: Wide pH gradients up to pH 12, longer separation distances and simplified procedures. Electrophoresis 20(4/5): 712–717. 10344237 10.1002/(SICI)1522-2683(19990101)20:4/5<712::AID-ELPS712>3.0.CO;2-Y

[15] Apel K, Hirt H (2004) Reactive oxygen species: metabolism, oxidative stress, and signal transduction. Annual Review of Plant Biology 55: 373–399 10.1146/annurev.arplant.55.031903.141701 15377225

[16] Kreps JA, Wu Y, Chang H, Zhu T, Wang X, et al (2002) Transcriptome changes for Arabidopsis in response to salt, osmotic, and cold stress. Plant Physiol 130: 2129–2141 10.1104/pp.008532 12481097 PMC166725

[17] Seki M, Narusaka M, Ishida J, Nanjo T, Fujita M, et al (2002) Monitoring the expression profiles of 7000 Arabidopsis genes under drought, cold and high-salinity stresses using a full-length cDNA microarray. Plant J 31: 279–292 10.1046/j.1365313X.2002.01359x 12164808

[18] Jiang YQ, Deyholos MK (2006) Comprehensive transcriptional profiling of NaCl-stressed Arabidopsis roots reveals novel classes of responsive genes. BMC Plant Biology 6: 25 10.1186/14712229625 17038189 PMC1621065

[19] Chen YM, Chen T, Shen SH, Zheng MZ, Guo YM, et al (2006) Differential display proteomic analysis of Picea meyeri pollen germination and pollen-tube growth after inhibition of actin polymerization by latrunculin B. The Plant Journal. 47: 174–195 10.1111/j.1365313X.2006.02783x 16771841

[20] Dhugga KS, Tiwari SC, Ray PM (1997) A reversibly gly-cosylated polypeptide (RGP 1) possibly involved in plant cell wall synthesis: purification, gene cloning, and trans-Golgi localization. Proc. Natl Acad Sci USA 94: 7679–7684. 10.1073/pnas.94.14.7679 PMC23882 9207152

[21] Sherrier DJ, VandenBosch KA (1994) Secretion of cell wall polysaccharides in Vicia root hairs. Plant J 5: 185–195.

[22] Hogetsu T, Shibaoka H (1978) Effects of colchicine on cell shape and on microfibril arrangement in the cell wall of Closterium acerosum. Biomedical and Life Sciences 1, 15–18. 10.1007/BF00389374 24414355

[23] Wang ZF, Wang ZH, Shi L, Wang LJ, Xu FS (2010) Proteomic alterations of Brassica napus root in response to boron deficiency. Plant Mol Biol 74: 265–278 10.1007/s111030109671-y 20694506

[24] Taylor NL, Heazlewood JL, Day DA, Millar AH (2005) Differential Impact of Environmental Stresses on the Pea Mitochondrial Proteome. Mol Cell Proteomics 4(8): 1122–1133 10.1074/mcp.M400210MCP200 15914488

[25] Lee H, Guo Y, Ohta M, Xiong L, Stevenson B, et al (2002) LOS_2_, a genetic locus required for cold-responsive gene transcription encodes a bi-functional enolase. EMBO J 21: 2692–2702 10.1093/emboj/21.11.2692 12032082 PMC126021

[26] Jiang YQ, Yang B, Harrisand NS, Deyholos MK (2007) Comparative proteomic analysis of NaCl stress-responsive proteins in Arabidopsis roots. J Exp Bot 13: 3591–3607 10.1093/jxb/erm207 17916636

[27] Parker R, Flowers TJ, Moore1AL, Harpham NVJ (2006) An accurate and reproducible method for proteome frofiling of the effects of salt stress in the rice leaf lamina. J Exp Bot 57(5): 1109–1118 10.1093/jxb/erj134 16513811

[28] Cui S, Huang F, Wang J, Ma X, Cheng Y, et al (2005) A proteomix analysis of cold stress responses in rice seedings. Proteomics 5(12): 3162–3172 10.1002/pmic.200401148 16078185

[29] Yan SP, Zhang QY, Tang ZC, Su WA, Sun WN (2006) Comparative proteomic analysis provides new insights into chilling stress responses in rice. Mol Cell Proteomics 5(3): 484–496 10.1074/mcp.M500251MCP200 16316980

[30] Tunc-Ozdemir M, Miller G, Song LH, Kim J, Sodek A, et al (2009) Thiamin Confers Enhanced Tolerance to Oxidative Stressin Arabidopsis. Plant Physiol 151: 421–432 10.1104/109.140046 19641031 PMC2735988

[31] Beale SI, Casteffranco PA (1974) The biosynthesis of delta-aminolevulinic acid in higher plants. II. Formation of 14C 8-aminolevulinic acid from labeled precursors in greening plant tissues. Plant Physiol 53: 297–303 10.1104/pp.53.2.297 16658694 PMC541382

[32] Wang JY (2003) Biochemistry (3th Edition). Beijing, China: Higher Education Press.

[33] Dixon DP, Lapthorn A, Edwards R (2002) Plant glutathione transferases. Genome Biol 3: 1–10. 10.1186/gb-2002-3-3-reviews3004 PMC139027 11897031

[34] Marrs KA (1996) The functions and regulation of glutathione S-transferases in plants. Annu Rev Plant Physiol, Plant Mol Biol 47: 127–158 10.1146/annurev.arplant.47.1.127 15012285

[35] Riechers DE, Irzyk GP, Jones SS, Fuerst EP (1997) Partial characterization of glutathione S-transferases from wheat (Triticum spp.) and purification of a safener-induced glutathione S-transferase from Triticum tauschii. Plant Physiol 114: 1461–1470 10.1104/pp.114.4.1461 9276955 PMC158439

[36] Cummins I, Cole DJ, Edwards R (1997) Purification of Multiple Glutathione Transferases Involved in Herbicide Detoxification from Wheat (Triticum aestivumL.) Treated with the Safener Fenchlorazole-ethyl. Pestic Biochem Physiol 59: 35–49.

[37] Dixon D, Cole DJ, Edwards R (1997) Purification of Multiple Glutathione Transferases Involved in Herbicide Detoxification from Wheat (Triticum aestivumL.) Treated with the Safener Fenchlorazole-ethyl. Pestic Biochem Phys 50: 72–82.

[38] Jepson I, Lay VJ, Holt DC, Bright SWJ, Greenland AJ (1994) Cloning and characterization of maize herbicide safener-induced cDNAs encoding subunits of glutathione S-transferase isoforms I, II and IV. Plant Mol Biol 26: 1855–1866 10.1007/BF00019498 7858222

[39] Davies J, Caseley JC (1999) Herbicide safeners: a review. Pestic Sci 55, 1043–1058.

[40] Xu FX, Lagudah ES, Moose SP, Riechers DE (2002) Tandemly duplicated safener-induced glutathione S-transferase genes from Triticum tauschii contribute to genome-and organ-specific expression in hexaploid wheat. Plant Physiol 130: 362–373 10.1104/pp.004796 12226515 PMC166568

[41] Edwards R, Dixon DP, Walbot V (2000) Plant glutathione S-transferases: enzymes with multiple functions in sickness and in health. Trends Plant Sci 5: 193–198 10.1016/S13601385(00)016010 10785664

[42] Ruiz Gómez MJ, Gil L, Souviron A, Martínez Morillo M (2000) Multidrug resistance increment in a human colon carcinoma cell line by colchicine. J Physiol Biochem 56 1: 33–38. 10.1007/BF03179774 10879679

[43] Teixeira FK, Menezes-Benavente L, Galvao VC, Margis R, Margis-Pinheiro M (2006) Rice ascorbate peroxidase gene family encodes functionally diverse isoforms localized in different subcellular compartments. Planta 224(2): 300–314 10.1007/s0042500502148 16397796

[44] Jung YH, Rakwal R, Agrawal GK, Shibato J, Kim JA, et al (2006) Differential expression of defense/stress-related marker proteins in leaves of a unique rice blast lesion mimic mutant (blm). J Proteome Res 5(10): 2586–2598 10.1021/pr060092c 17022630

[45] Salekdeh GH, Siopongco J, Wade LJ, Ghareyazie B, Bennett J (2002) proteomic analysis of rice leaves during droughtstress and recovery. Proteomics 2(9): 1131–1145 10.1002/16159861 (200209).. 12362332

[46] Agrawal GK, Rakwal R, Yonekura M, Kubo A, Saji H (2001) Proteome analysis of differentially displayed proteins as a tool for investigating ozone stress in rice (Oryza sativa L.) seedlings. Proteomics 2(8): 947–959 doi: –;10.1002/1615–9861(200208)2:8<947:: AID-PROT947>3.0.CO;2-J 10.1002/1615-9861(200208)2:8<947::AID-PROT947>3.0.CO;2-J 12203890

[47] Agrawal GK, Rakwal R, Tamogami S, Yonekura M, Kubo A, et al (2002) Chitosan activates defense/stress response(s) in the leaves of Oryza sativa seedlings. Plant Physiology and Biochemistry 40(12): 1061–1069.

[48] Drążkiewicz M, Skórzyńska-Polit E, Wanke M, Świeżewska E (2003) The activity of antioxidant enzymes in arabidopsis thaliana exposed to colchicine and H_2_O_2_ . Cellular & Molecular Biology Letters 8: 777–781. 12949616

[49] Shin M, Arnon DI (1965) Enzymic mechanisms of pyridine nucleotide reduction in chloroplasts. J Biol Chem 240: 1405–1411. 14284756

[50] Krapp AR, Carrillo N (1995) Functional complementation of the mvrA mutation of Escherichiu coli by plant ferredoxin-NADP^+^ -oxidoreductase. Arch Biochem Biophys 317: 215–221. 7872787 10.1006/abbi.1995.1156

[51] Coux O, Tanaka K, Goldberg AL (1996) Structure and functions of the 20S and 26S proteasomes. Annu Rev Bioche 65: 801–847 10.1146/annurev.bi.65.070196.004101 8811196

[52] DeMartino GN, Moomaw CR, Zagnitko OP, Proske RJ, Ma CP, et al (1994) PA700, an ATP-dependent activator of the 20 S proteasome, is an ATPase containing multiple members of a nucleotide-binding protein family. J Biol Chem 269: 20878–20884. 8063704

[53] Dubiel W, Ferrell K, Rechsteiner M (1995) Subunits of the regulatory complex of the 26S protease. Mol Biol Rep 21: 27–34 10.1007/BF00990967 7565660

[54] Genschik P, Parmentier Y, Durr A, Marbach J, Criqui MC, et al (1992) Ubiquitin genes are differentially regulated in protoplast-derived cultures of Nicotiana sylestris and in response to various stresses. Plant Mol Biol 20: 897–910 10.1007/BF00027161 1281439

[55] Feussner K, Feussner I, Leopold I, Wasternack C (1997) Isolation of a cDNA coding for an ubiquitin-conjugating enzyme UBC1 of tomato – the first stress-induced UBC of higher plants. FEBS Lett 409: 211–215 10.1016/S0014-5793(97)005097 9202147

[56] Chevalier C, Le Querrec F, Raymond P (1996) Sugar levels regulate the expression of ribosomal protein genes encoding protein S28 and ubiquitin-fused protein S27a in maize primary root tips. Plant Sci 117: 95–105.

[57] Sun CW, Callis J (1997) Independent modulation of Arabidopsis thaliana polyubiquitin mRNAs in different organs and in response to environmental changes. Plant J 11: 1017–1027 10.1046/j.1365313X.1997.11051017x 9193073

[58] Wegener A, Gimbel W, Werner T, Hani J, Ernst D, et al (1997) Sequence analysis and ozone-induced accumulation of polyubiquitin mRNA in Pinus sylestris. Can J For Res 27: 945–948.

[59] Brosche M, Fant C, Bergkvist SW, Strid H, Svensk A, et al (1999) Molecular markers for UV-B stress in plants: Alteration of the expression of four classes of genes in Pisum satium and the formation of high molecular mass RNA adducts. Biochem Biophys Acta 1447: 185–198 10.1016/S01674781(99)001542 10542315

[60] Wanke M, Dallner G, Świeżewska E (2000) Subcellular localization of plastoquinone and ubiquinone biosynthesis in spinach cells. BBA-Biomembranes 1463(1): 188–194. 10631308 10.1016/s0005-2736(99)00191-1

[61] Dou F, Netzer WJ, Tanemura K, Li F, Hartl FU, et al (2003) Chaperones increase association of tau protein with microtubules. Proc. Natl Acad Sci USA 100: 721–726 10.1073/pnas.242720499 PMC141063 12522269

[62] Durso NA, Cyr RJ (1994) A calmodulin-sensitive interaction between microtubules and a higher plant homolog of elongation factor-1a. Plant Cell 6: 893–905. 8061523 10.1105/tpc.6.6.893 PMC160487

[63] Olmsted JB (1991) Microtubule-associated proteins. Curr. Opin. Cell Biol 3: 52–58. 10.1016/0955-0674(91)90165-u 1854484

[64] Chapin SJ, Bulinski JC (1992) Microtubule stabilization by assembly-promoting microtubule-associated proteins: A repeat performance. Cell Motil Cytoskeleton 23: 236–243 10.1002/cm.970230403 1477887

[65] Hirokawa N (1994) Microtubule organization and dynamics dependent on microtubule-associated proteins. Curr Opin Cell Biol 6: 74–81 10.1016/09550674(94)901198 8167029

[66] Drechsel DN, Hyman AA, Cobb MH, Kirschner MW (1992) Modulation of the dynamic instability of tubulin assembly by the microtubule-associated protein tau. Mol Biol Cell 3: 1141–1154. 1421571 10.1091/mbc.3.10.1141 PMC275678

[67] Pryer NK, Walker RA, Skeen VP, Bourns BD, Soboeiro MF, et al (1992) Brain microtubule-associated proteins modulate microtubule dynamic instability in vitro: Realtime observations using video microscopy. J Cell Sci 103: 965–976. 1487507 10.1242/jcs.103.4.965

[68] Gamblin TC, Nachmanoff K, Halpain S, Williams RC Jr (1996) Recombinant microtubule-associated protein 2c reduces the dynamic instability of individual microtubules. Biochemistry 35: 12576–12586 10.1021/bi961135d 8823195

[69] Gard DL, Kirschner MW (1987) A microtubule-associated protein from Xenopus eggs that specifically promotes assembly at the plusend. J Cell Biol 105: 2203–2215 10.1083/jcb.105.5.2203 2890645 PMC2114854

[70] Vasquez RJ, Gard DL, Cassimeris L (1994) XMAP from Xenopus eggs promotes rapid plus end assembly of microtubules and rapid microtubule polymer turnover. J Cell Biol 127: 985–993 10.1083/jcb.127.4.985 7962080 PMC2200056

[71] Dhamodharan R, Wadsworth P (1995) Modulation of microtubule dynamic instability in vivo by brain microtubule associated proteins. J Cell Sci 108: 1679–1689. 7615685 10.1242/jcs.108.4.1679

